# Semantic memory as the root of imagination

**DOI:** 10.3389/fpsyg.2015.00325

**Published:** 2015-03-24

**Authors:** Anna Abraham, Andreja Bubic

**Affiliations:** ^1^School of Social, Psychological and Communication Sciences, Faculty of Health and Social Sciences, Leeds Beckett UniversityLeeds, UK; ^2^Psychology Department, Faculty of Humanities and Social Sciences, University of SplitSplit, Croatia

**Keywords:** creativity, prospection, neurocognition, cognitive neuroscience, mental time travel, episodic memory, theory of mind, moral reasoning

“Imagination is what makes our sensory experience meaningful, enabling us to interpret and make sense of it, whether from a conventional perspective or from a fresh, original, individual one. It is what makes perception more than the mere physical stimulation of sense organs. It also produces mental imagery, visual and otherwise, which is what makes it possible for us to think outside the confines of our present perceptual reality, to consider memories of the past and possibilities for the future, and to weigh alternatives against one another. Thus, imagination makes possible all our thinking about what is, what has been, and, perhaps most important, what might be.”—Nigel J. T. Thomas (2004, as cited in Manu, 2006, p. 47)^1^.

Investigations of the information processing mechanisms that underlie imaginative thought typically focus on a single branch of imagination, such as prospection, mental imagery or creativity, and are often generalized as being insightful to understanding the workings of imagination in general. In reality, however, there is very little in the way of theoretical or empirical exchange between the scientific communities that conduct research within the different domains of imagination. As a result, the research impetus in each of the sub-domains may be skewed to the pursuit of hypotheses that are not particularly viable in terms of understanding imagination as a whole. An example of this is pegging the roots of imagination to the processes of episodic memory—a reasonable assumption to make based on studies of episodic prospection. However, the associated findings and theoretical conclusions that follow are not entirely consistent with the literature on the mechanisms underlying creativity (Bubić and Abraham, 2014), which is another core realm of imagination.

In an effort to promote interchange across the frontiers of imagination, in this Opinion Article we put forward the idea that all aspects of imagination emerge from semantic memory with increasingly higher-order levels of imaginative information processing emanating from and interacting with existing systems, eventually expanding beyond these to form new systems (Figure 1). We compare the associated neurocognitive findings and assumptions in terms of their fit with current knowledge in other fields of imagination and discuss their implications for reformulating hypotheses regarding imagination as a whole.

**Figure 1:**
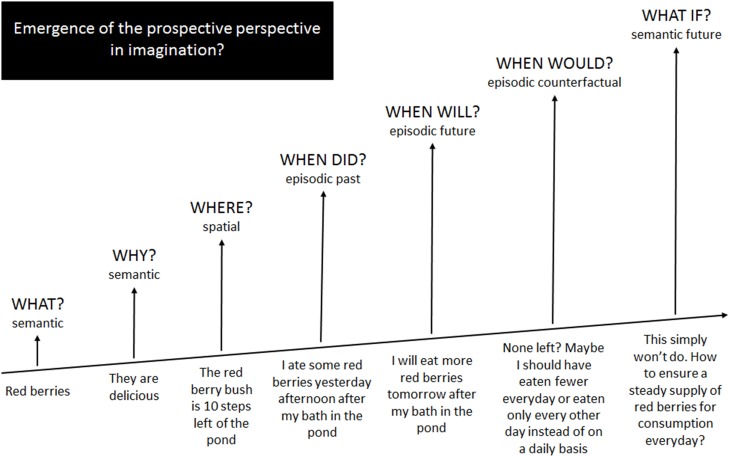
**An informal illustration of how imaginative processes emerge from and expand beyond semantic memory operations**.

## The what?

Our conceptual knowledge of the world is the foundation from which all imaginative thought emerges and, as such, constitutes “the what-system” within the information processing hub. Investigations of the manner in which concepts are acquired, represented, stored, and accessed fall within the field of semantic cognition. The brain networks that underlie the what-system include modality-specific sensory and motor systems as well as multimodal or supramodal regions within the inferior parietal lobe, middle and inferior temporal gyri, fusiform and parahippocampal gyri, inferior frontal gyrus, dorsomedial and ventromedial prefrontal cortex and the posterior cingulate gyrus (Binder et al., 2009; Binder and Desai, 2011; Kiefer and Pulvermüller, 2012). Such insights have emerged from neuroscientific investigations into the brain basis of semantic memory, semantic aspects of language processing, and the organization of conceptual knowledge in the brain.

## The what–where?

Determining the location of any object or person relative to oneself, some other person or object is only possible by accessing representations of spatial information such as direction, orientation, distance and position of that object or person. Such information is coded by means of reference frames relative to the observer (egocentric) and independent of the observer (allocentric) (Burgess, 2006). Tasks of spatial memory and navigation have shown that medial temporal lobe structures such as the hippocampal formation, parahippocampal gyrus, entorhinal and perirhinal cortices as well as medial parietal regions, such as the retrosplenial and posterior cingulate cortices (Burgess, 2008; Chrastil, 2013; Ekstrom et al., 2014), are critically involved in spatial information processing. Others tasks of spatial cognition, such as perspective taking, have indicated the involvement of additional regions within the posterior parietal cortex, particularly the inferior parietal and temporo-parietal areas (Byrne and Becker, 2007; Dhindsa et al., 2014).

## The what–where–when?

An event is defined as a specific happening (what) that occurs at a certain place (where) and at a given time (when). During retrospection we access events from our personal past (episodic memory, autobiographical memory), whereas during prospection we contemplate events that could unfold in our personal future (episodic future thinking). Both fall within the umbrella concept of mental time travel (Tulving, 1985). Neuroscientific evidence has consistently revealed that the brain network that is engaged when we imagine personal events in the near or distant future overlaps considerably with the network that is activated when we ponder our episodic or autobiographical past (Schacter et al., 2007, 2012; Mullally and Maguire, 2013). Regions that comprise this brain network include the ventral and dorsal medial prefrontal cortex, retrosplenial and posterior cingulate regions within the medial parietal cortex, anterior lateral temporal cortex, inferior parietal cortex, and medial temporal lobe structures such as the hippocampus. Notably, the regions of the mental time travel brain network also closely correspond to those of the brain's default mode network (DMN). The DMN is active under conditions of rest and low task load, and is held to reflect processing demands associated with mind-wandering, internal mentation and stimulus-independent thought (Andrews-Hanna et al., 2014). DMN brain areas are also involved in other facets of higher order cognition, like mental state reasoning or theory of mind, moral cognition, and self-referential thought (Buckner et al., 2008; Spreng et al., 2009) all of which involve reasoning about one's self and/or others.

## The what-if?

Our capacity to imagine possibilities is virtually unconstrained. Investigations on the information processing circuits involved in prospection address the question of “what if?” or “what might be?” within a specific temporal context of our personal lives (which covers the aforementioned episodic prospection of the what–when–where system). However, our cognitive capacity to explore hypothetical possibility spaces is neither limited only to our personal lives nor to any temporal factor (past/present/future). Other operations that fall under the category of what-if or hypothetical reasoning based cognitive processes include semantic prospection, semantic or episodic counterfactual reasoning and creativity. In addition to the partial conceptual overlap between the what-if system and the previously discussed what-where-when system, the two also share common underlying neural mechanisms. Although only a few neuroscientific studies have investigated semantic prospection or the propensity to think about the non-personal future, the limited evidence indicates that semantic prospection is reliant on similar parts of the brain's episodic mental time travel network, particularly with reference to the engagement of anterior and dorsal medial prefrontal regions, inferior parietal cortices, the hippocampus and related medial temporal lobe structures (Abraham et al., 2008; Race et al., 2013).

In contrast to semantic prospection, which is relatively unrestricted with regard to the types of imaginable alternatives, counterfactual thinking primarily involves exploring possibilities that are contrary to what has already come to pass. Research on brain correlates of counterfactual comparisons and emotions that often accompany such cognition, such as regret, indicates a key role for the orbitofrontal and ventromedial prefrontal cortices (Camille et al., 2004) Furthermore, studies that have assessed episodic past, episodic future and episodic counterfactual thinking have reported a common brain network, involving the hippocampal formation, temporal lobe structures, lateral parietal regions as well as medial and lateral prefrontal areas. Within the episodic cognition domain, counterfactual thinking recruits some of these areas more strongly than past and future thinking, and also additionally engages the bilateral inferior parietal lobe and posterior medial frontal cortex (Van Hoeck et al., 2013).

Semantic prospection and counterfactual reasoning are concerned with hypothetical reasoning linked to the future and the past, respectfully. However, one can also engage in hypothetical reasoning within temporally unspecific contexts such as those involving moral and mental state reasoning, which, as pointed out earlier, strongly overlap in terms of their implicated brain network with the what–when–where system (Buckner et al., 2008). While the contexts tapped in such hypothetical reasoning operations are decidedly social in nature, a non-socially based avenue within which we necessarily exercise our capacity to think hypothetically is that of creativity.

Our capacity to be creative is examined by assessing the extent to which we are able to generate original and relevant responses to a particular end (Stein, 1953; Runco and Jaeger, 2012). The underlying brain mechanisms of creative cognition are very complex (Abraham, 2014). Brain regions such as the dorsal and ventral medial prefrontal cortex, retrosplenial and posterior cingulate cortices as well as medial temporal lobe structures are strongly engaged during divergent thinking, or the generation of multiple responses in an open-ended situation (Abraham et al., 2012) This indicates that there is a considerable overlap in the neural correlates of divergent thinking and that of the what–when–where network. While divergent thinking certainly involves hypothetical reasoning and exploration of an abstract possibility space, it does not necessarily translate to creative thought. Having constraints on divergent thinking pushes the information processing system to be necessarily creative (both original and relevant) and this leads to the additional activation of the semantic cognition and cognitive control networks with the major contributions being provided by brain regions such as the inferior frontal gyrus, temporal pole, frontopolar cortex, and basal ganglia. So the neural correlates of creative cognition system overlap only partially with those associated with other aspects of the imagination system with common activations seen in the dorsomedial prefrontal cortex and inferior parietal lobe (the what–when–where system) as well as the inferior frontal gyrus (the what-system).

## Integrating the disparate systems of imagination

In this Opinion Article, we explored the view that processes of imagination—the “where” of spatial cognition, the “what-when-where” of episodic retrospection and prospection, and the “what-if” of semantic prospection, counterfactual reasoning and creative thinking—emerge from a foundation provided by the “what” of semantic memory operations. The evidence thus far clearly indicates that the many processes of imagination, which have mostly been systematically investigated in isolation from one another, are neurally implemented in substantially overlapping brain networks and are also similar with respect to their underlying cognitive algorithms and mechanisms. This resonates with other proposals that have highlighted that semantic and episodic cognitive operations and their related brain systems are dynamically interlinked (Squire and Zola, 1998; Greenberg and Verfaellie, 2010), as well as with recent calls for de-emphasizing the episodic or autonoetic aspects of future oriented cognition and advocating the central role played by semantic memory in the same (Stocker, 2012; Irish and Piguet, 2013).

This does not mean that all imaginative processes are to be considered “atemporal” per definition. Many forms of mental time travel as well as counterfactual thinking patently involve the consideration of temporal factors as a core facet of the imaginative process. In taking this a step further, it may even be argued that such processes are necessarily linked to the brain's predictive systems due to the fact that they involve the generation of estimates concerning events that reliably unfold over a certain period of time, albeit with differing levels of certainty (Bubic et al., 2010). This position has rarely been considered in the literature on imagination-relevant operations but it would fit with a number of suggestions that posit prediction as the fundamental mechanism that modulates our general neural and cognitive processing (Friston and Stephan, 2007; Pezzulo, 2008).

So, although the issue of temporality is undoubtedly relevant, the more fundamental basis that underlies all of the aforementioned processes is the reliance on our experiences with the world, its objects and events. We therefore suggest that if the aim is to develop a comprehensive information processing model of imagination, the foundational elements should be discussed in terms of semantic memory operations. As semantic memory involves the abstraction of content from experiences that are specific to sensory, motor, or affective modalities, conceptualizing the processes of imagination as stemming from semantic operations allows for a more seamless integration of its theoretical models with that of the wider research realm of perception, action and cognition where concepts such as embodied cognition and predictive processing are revolutionizing our understanding of psychology.

We hope these ideas will stimulate future research and the development of novel paradigms as well as critical scientific exchange between the research communities involved in understanding different aspects of imagination. Some questions can be already anticipated such as the “chicken-and-egg” problem within which it appears impossible to clearly substantiate what came first, or concerns about how to reach a consensus about what can be considered an underlying foundational element. Through the process of this discussion though, we hope that building blocks and essential frameworks will be uncovered that will guide us through the incredibly rich world of human imagination.

### Conflict of interest statement

The authors declare that the research was conducted in the absence of any commercial or financial relationships that could be construed as a potential conflict of interest.

## Footnotes

^1^http://archive.today/www.imagery-imagination.com or http://www.co-bw.com/BrainConciousness%20Update%20index.htm
